# Globular adiponectin protects H9c2 cells from palmitate-induced apoptosis via Akt and ERK1/2 signaling pathways

**DOI:** 10.1186/1476-511X-11-135

**Published:** 2012-10-11

**Authors:** Chuan Dong Wei, Yan Li, Hong Yun Zheng, Kai Sheng Sun, Yong Qing Tong, Wen Dai, Wei Wu, An Yu Bao

**Affiliations:** 1 Department of Clinical Laboratory, Renmin Hospital of Wuhan University, 99 Ziyang Road, District of Wuchang, Wuhan, Hubei, 430060, China; 2 Department of Clinical Laboratory, Affiliated Hospital of Youjiang Medical University for nationalities, Baise, Guangxi, 533000, China

**Keywords:** Adiponectin, H9c2 cells, Palmitate, Apoptosis, PI3K/Akt, ERK1/2

## Abstract

**Background:**

Cardiomyocytes apoptosis is an important contributor to myocardial dysfunction and heart failure. Adiponectin has cardioprotective effects, potential mechanisms behind it are not clear in cardiomyocytes. The purpose of the study was to investigate whether adiponectin can block palmitate-induced apoptosis and the underlying biochemical mechanism in H9c2 cells.

**Methods:**

H9c2 cells were treated with palmitate presence or absence of 2.5 μg/mL globular adiponectin. The effect on the cell viability of H9c2 cells was evaluated using MTT assay, and cell apoptosis was determined by Hoechst 33342 staining. Protein expression was measured using the western blot method.

**Results:**

Our results showed that the palmitate treatment induced apoptosis in H9c2 cells, which was associated with increasing the level of cleaved caspase-3 and cleaved PARP. Meanwhile, palmitate-induced apoptosis increased the protein level of p-ERK1/2, and decreased the protein level of p-Akt significantly. However, levels of both of these proteins were restored to the normal when pretreated with adiponectin, and followed with the decrease of cleaved caspase-3 and cleaved PARP. In line with these results, the protective effect of adiponectin can be blocked by PI3K/Akt inhibitor LY294002, and palmitate-induced apoptosis can be attenuated by ERK1/2 inhibitor U0126.

**Conclusions:**

Taken together, the present study demonstrated that adiponectin protects H9c2 cells from palmitate-induced apoptosis via PI3K/Akt and ERK1/2 signaling pathways. Our results reveal a link between adiponectin and cardiomyocytes apoptosis, suggesting that adioponectin may be a promising therapeutic for the treatment of lipotoxicity cardiomyopathy.

## Background

Lipids over accumulation in the heart are associated with cardiac dysfunction and heart failure [1,2]. Saturated fatty acid such as palmitate (C16:0) but not monounsaturated oleate (C18:1) induced apoptosis in many cell types including the cardiomyocytes element of the heart [3]. Several studies showed that high levels of palmitate had caused significant increases of ceramide and mitochondrial cytochrome c release levels, which was accompanied by significant caspase-3 activation and apoptosis [4,5], and this lipotoxicity effect on cardiac myocytes apoptosis remains incompletely understood. Cardiomyocytes apoptosis is an important contributor to myocardial dysfunction and heart failure, and blockade of myocardial apoptosis results in significant prevention of diabetes-induced cardiac dysfunction [6].

Adiponectin is an adipokine secreted from adipose tissue and plasma with concentrations ranging from 3 to 30 μg/mL in mouse and human [7,8]. The structure of adiponectin contains a collagen-repeat domain at the N-terminus and a globular domain at the C-terminus with a sequence homology to compliment factor C1q [8]. The C-terminal globular C1q domain of adiponectin is proteolytically cleaved from the full length protein and is also able to circulate in both human and mouse plasma to mediate potent physiological effects [9,10]. Recently, a number of studies have shown that adiponectin transcripts are synthesized by cardiomyocytes [11], which are upregulated in mouse models of myocardial injury [12,13]. To date, adiponectin has been extensively documented to mediate several cardioprotective properties [14], and anti-apoptotic effect of adiponectin on the heart [12,13,15].

Phosphatidylinositol 3-kinase (PI3K)/Akt and extracellular signaling regulated kinase (ERK1/2)/mitogen-activated protein kinase (MAPK) signaling pathways are an important for intracellular signal transduction system. PI3K/Akt has been shown to play a major role in the prevention of apoptosis [16], and ERK1/2 is a well-known taking part in a signal transduction cascade in response to extracellular stimuli, and plays an important role in cell proliferation, growth and cell death [17]. Several studies have exhibited that anti-apoptotic effect of adiponectin on the heart, which appeared to be mediated via PI3K/Akt, ERK1/2MAPK and AMP-activated protein kinase (AMPK) signaling pathway [15,18,19]. Adiponectin could protect against acute cardiac injury by attenuating the apoptosis, but the mechanism involved the effect of adiponectin in palmitate-induced apoptosis are not fully understood.

In the present study, we demonstrated that adiponectin protected H9c2 cells from palmitate-induced apoptosis through both PI3K/Akt and ERK1/2 signaling pathways.

## Materials and methods

### Chemicals and reagents

Dulbecco’s Modified Eagle Medium (DMEM) and Penicillin/Streptomycin were obtained from Thermo Scientific Hyclone (Hyclone, US). Rat recombinant globular adiponectin (gAd) was purchased from Biovision (Biov, USA). Antibodies for the phosphorylated Akt at Ser473, total-Akt, cleaved caspase-3, poly (ADP-ribose) polymerase (PARP), inhibitor of PI3K LY29002, inhibitor of p-ERK1/2 U0126, HRP-conjugated anti-rabbit or anti mouse secondary antibodies were obtained from Cell Signaling Technology (Cell signaling, USA). Antibodies for the phosphorylated at Thr202/Tyr204 extracellular-regulated kinase (p-ERK1/2), total ERK1/2, β-actin, and Enhanced chemiluminescence (ECL) reagent were purchased from Millipore (Millipore, USA). Palmitate (PA) and Bovine Serum Albumin (BSA) were obtained from Sigma Aldrich (Sigma, USA). The stock solutions 5 mM PA/10% BSA that can be stored at −20°C was prepared reference from [20]. The 5 mM PA/10% BSA stock solutions are heated for 15 min at 55°C, and then cooled to room temperature before use.

### Cell culture

H9c2 cells (a subclone of the original clonal cell line derived from embryonic BD1X rat heart tissue exhibiting many of the properties of skeletal muscle) obtained from Chinese Collection of Cell Cultures, were grown in Dulbecco’s Modified Eagle’s Medium (DMEM) supplemented with 10% fetal bovine serum and 1% penicillin–streptomycin in a humidified atmosphere of 95% air-5% CO_2_ at 37°C. In addition, the various treatments for cells were carried out only when cells reach about 80% of confluence in appropriate culture dish.

### Nuclear staining with Hoechst 33342

Cell were plated in 6 well chamber slides and allowed to adhere. Following 12 h different treatment, cells from each group were washed with phosphate buffered saline and fixed with 4% formalin for 10 min. Hoechst 33342 (10 μg/mL) was applied for 30 min under dark condition to stain the nuclei of fixed cells. Slides were then washed with phosphate buffered saline and mounted in a mounting medium (phosphate buffered saline: glycerol, 1:1), and observed under a fluorescence microscope. Apoptotic cells were identified as those with a nucleus exhibiting brightly stained condensed chromatin or unclear fragments. For each experimental condition, four separate cell populations were prepared. Apoptotic indices were determined by direct visualization and counting of a minimum of 500 cells per population (at least 100 cells from five randomly selected fields). The apoptotic index was calculated as the ratio of number of apoptotic cells to total cells counted × 100.

### Cell viability assay

Cell viability was measured using the MTT (3-(4,5)-dimethylthiahiazo (−z-y1)-3,5-diphenytetrazoliumromid) assay, based on the MTT conversion into formazan crystals using mitochondrial dehydrogenases. Briefly, H9c2 cells were plated at a density of 1 × 10^4^ cells/well in 96-well plates. After different treatment for 12 h, the culture medium was replaced with 200 μL MTT solution (5 mg/mL stock solution in PBS, diluted with culture medium to the final concentration 0.5 mg/mL). After 4 h incubation at 37°C, this solution was removed and the produced formazan was solubilized in 150 μL dimethyl sulfoxide (DMSO). The absorbance was measured at 550 nm using an automated microplate reader.

### Immunoblot

Cells were lysed in ice-cold RIPA buffer (50 μM Tris/HCl, pH 7.4, 150 μM NaCl, 1% NP-40, 0.5% Deoxycholate, 0.1% Sodium dodecyl sulfate) and the protease of inhibitor phenylmethanesulfonyl fluoride (PMSF). Protein concentration of the cell samples was determined using the bicinchoninic acid protein assay reagent kit (Pierce Biotechnology, USA) with bovine serum albumin as standard. For Western blot analysis, 40 μg of protein was denatured by heating 100°C for 10 min in SDS sample buffer, loaded onto and separated by 10% or 12% SDS polyacrylamide gels, and then transferred electrically to a polyvinylidene fluoride (PVDF) membrane. The membrane was blocked in 5% (wt/vol) nonfat milk with 0.05% Tween-20 TBS buffer for 1 h and then was incubated overnight with the following different primary antibodies: monoclonal anti-Akt (1:1000) and anti-p-Akt (ser473) (1:1000), monoclonal anti-cleave caspase-3 (1:500), monoclonal anti-PARP (1:1000), monoclonal anti-p-ERK1/2 (Thr202/Tyr204) (1:500), monoclonal anti-ERK1/2 (1:500), and anti-β-actin antibody (1:2000) was used to show equal loading of the protein in the western blotting and quantitative analysis. The membranes were incubated with horseradish peroxidase-linked anti-mouse or anti-rabbit secondary antibody at 1:3000 dilutions for 1 h at 37°C and, after washes, visualized for immunoreactivity using an Enhanced Chemiluminescence (ECL) System.

### Statistical analysis

Quantitative data are presented as the means ± SE determined from at least three independent of experiments. Statistical analysis was based on Student’s *t* test for comparison of two groups or one-way ANOVA for multiple comparisons. *P* value <0.05 was considered significant.

## Results

### Palmitate-induced H9c2 cells apoptosis through activation of caspase-3 and PARP

**Figure 1:**
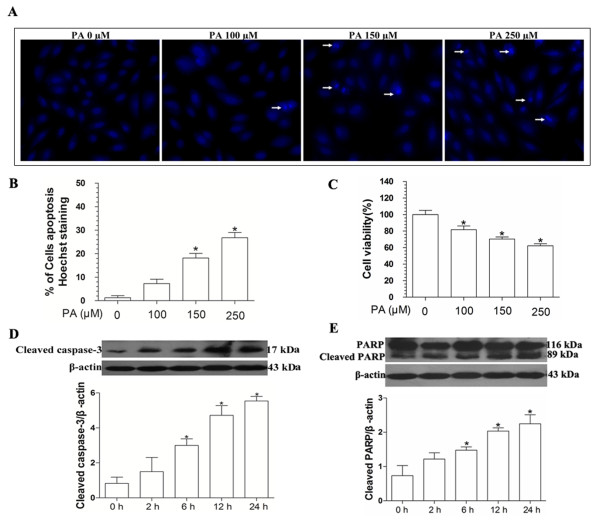
**Palmitate-induced H9c2 cells apoptosis.** Cells were treated with different concentrations of palmitate (0 μM, 100 μM, 150 μM and 250 μM) for 12 h, cell apoptosis was determined by Hoechst 33342 staining (**A** and **B**), and cell viability was measured using MTT assay (**C**). Cells were treated with 150 μM palmitate on time-dependent. Levels of cleaved caspase-3 (**D**) and PARP (**E**) were measured by immunoblot and quantitative analysis. These results were reported as mean ± S.E.M. * *P*< 0.05 versus control.

In order to determine the toxic effects of palmitate on H9c2 cells, cells were treated with increasing palmitate (PA) from 0 to 250 μM for 12 h. An increase in the number of apoptotic cells was observed in H9c2 cells by Hoechst 33342 staining (Figure 1A, B), and decreased cell viability was measured by a MTT assay (Figure 1C). Next, we choose the 150 μM palmitate in subsequent experiments to analyze cleaved caspase-3 and the cleavage of poly (ADP-ribose) polymerase (PARP), two well-established hallmarks of apoptosis [21,22]. Immunoblot and quantitative analysis results showed that expression of cleaved caspase-3 was detected at 2 h after treatment with 150 μM palmitate, and increased gradually at 6 h, 12 h and 24 h (Figure 1D). The analysis result of PARP cleavage (Figure 1E) was similar to that of the cleaved caspase-3. These results suggested that caspase-3 and PARP activation were involved in the apoptotic pathway induced by palmitate in H9c2 cells.

### Adiponectin attenuated palmitate-induced H9c2 cells apoptosis through reduced the activation of caspase-3 and PARP

**Figure 2:**
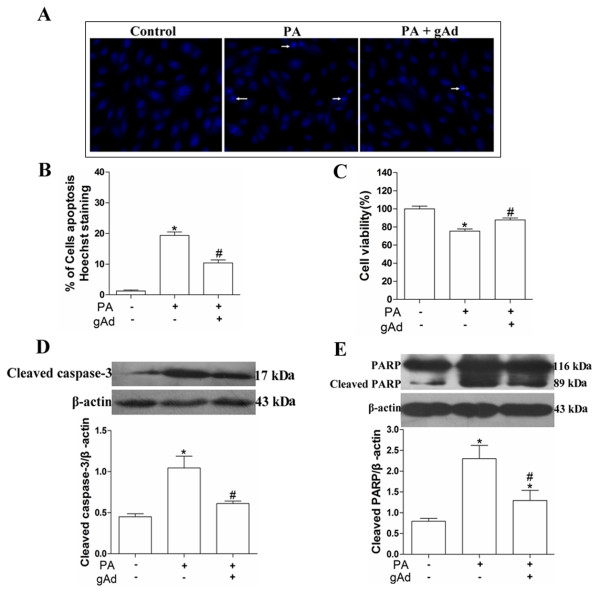
**Effects of adiponectin on palmitate**-**induced apoptosis in H9c2 cells.** Cells were pretreated with 2.5 μg/mL globular adiponectin for 1 h before exposure to 150 μM palmitate for 12 h. Cell apoptosis was determined by Hoechst 33342 staining (**A** and **B**), and cell viability was measured using MTT assay (**C**). Levels of cleaved caspase-3 (**D**) and PARP (**E**) were measured by immunoblot and quantitative analysis. Results were reported as mean ± S.E.M. * *P*< 0.05 versus control, # *P*< 0.05 versus PA.

Adiponectin exists in the circulation as a full-length protein and cleaved globular C-terminal domain, both of which are pharmacologically active [9]. In this study, there were three groups, 1% BSA control, palmitate (PA) treated group as well as globular adiponectin (gAd) and palmitate treated group, and the concentration of 2.5 μg/mL globular adiponectin was chosen reference from [23]. Cells were treated with 150 μM palmitate for 12 h or pretreated with 2.5 μg/mL globular adiponectin for 1 h and then treated with 150 μM palmitate for 12 h. BSA treated cells were used as the control. After the treatment, apoptosis of cell was measured using Hoechst 33342 staining, viability of cells was measured by a MTT assay; and expression of cleaved caspase-3 and cleaved PARP were measured by immunoblot. Results showed that apoptosis of cells increased (Figure 2A, B), viability of cells reduced (Figure 2C), levels of cleaved caspase-3 and cleaved PARP increased significantly after treated with palmitate (Figure 2D, E). However, when pretreated with adiponectin, we found that adiponectin pretreatment significantly decreased apoptosis of cells (Figure 2A, B), increased viability of cells (Figure 2C), reduced the level of cleaved caspase-3 (Figure 2D) as well as cleaved PARP (Figure 2E). These results indicated that adiponectin might attenuate palmitate-induced apoptosis in H9c2 cells through reducing the activation of caspase-3 and PARP.

### PI3K/Akt was involved in the process of adiponectin-mediated anti-apoptosis

**Figure 3:**
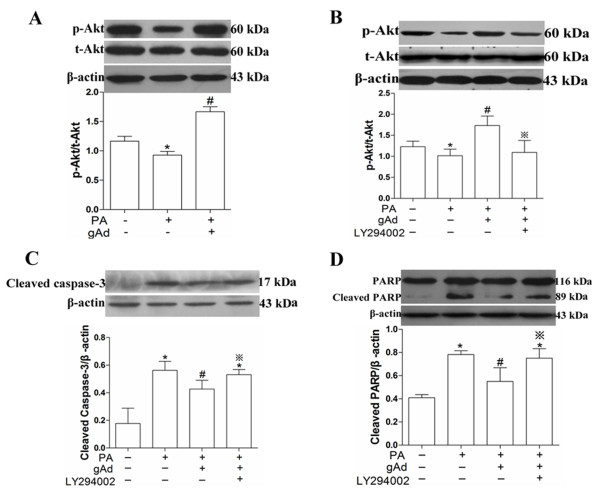
**PI3K**/**Akt signaling pathway was involved in the process of adiponectin**’**s anti**-**apoptosis in H9c2 cells.** Cells were pretreated with 2.5 μg/mL globular adiponectin for 1 h, in the presence or absence of PI3K inhibitor, LY294002, and then exposure to 150 μM palmitate for 12 h. Levels of p-Akt (**A** and **B**), t-Akt (**A** and **B**), cleaved caspase-3 (**C**) and PARP (**D**) were measured by immunoblot and quantitative analysis. These results were reported as mean ± S.E.M. * *P*< 0.05 versus control, # *P*< 0.05 versus PA and ※ *P*< 0.05 versus (PA + gAd).

Adiponectin is also known to activate PI3K/Akt signaling pathway, and the involvement of this signaling pathway in suppressive effects of adiponectin on palmitate-induced apoptosis was investigated by PI3K inhibitor, LY294002. The level of p-Akt was decreased after exposure of H9c2 cells to palmitate for 12 h (Figure 3A). Simultaneously the level of cleaved caspase-3 and cleaved PARP was increased significantly (Figures 2C, D). Cells were first pretreated with 2.5 μg/mL globular adiponectin, then treated with palmitate for 12 h, and lastly assayed by immunoblot. Results showed that the level of p-Akt decreased dramatically after treated with palmitate. However, its level restored to the control level after pretreated with 2.5 μg/mL globular adiponectin (Figure 3A). To test whether PI3K/Akt signaling pathway was involved in the inhibitory effect of adiponectin on palmitate-induced apoptosis in H9c2 cells, we used the inhibitor of PI3K/Akt, 10 μM LY294002 reference from [24]. Cells were first pretreated with 10 μM LY294002 for 1 h, then treated with 2.5 μg/mL globular adiponectin for another 1 h, and lastly treated with palmitate for 12 h. Results showed that the restored level of p-Akt induced by 2.5 μg/mL globular adiponectin was decreased again (Figure 3B), and levels of cleaved caspase-3 and cleaved PARP were also reversed after pretreated with LY294002 compared with 2.5 μg/mL globular adiponectin plus palmitate group (Figure 3C, D). Taken together, these results demonstrated that adiponectin partially inhibited palmitate-induced apoptosis in H9c2 cells via activating the PI3K/Akt signaling pathway.

### ERK1/2 was also involved in the process of adiponectin-mediated anti-apoptosis

**Figure 4:**
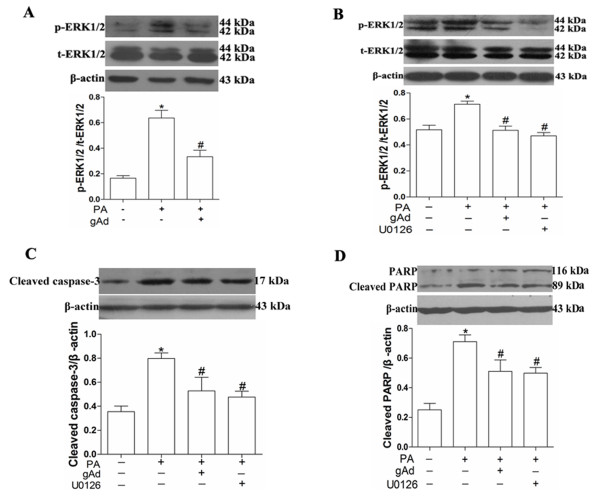
**ERK1**/**2 signaling pathway was also involved in the process of adiponectin**’**s anti**-**apoptosis in H9c2 cells.** Cells first were pretreated with 2.5 μg/mL globular adiponectin for 1 h, in the presence or absence of ERK1/2 inhibitor, U0126 (10 μM), and then exposed to 150 μM palmitate for 12 h. Levels of p-ERK1/2 (**A** and **B**), total-ERK1/2 (**A** and **B**), cleaved caspase-3 (**C**) and PARP (**D**) were measured by immunoblot and quantitative analysis. Results were reported as mean ± S.E.M. * *P*< 0.05 versus control, # *P*< 0.05 versus PA.

In the present study, results showed that the level of p-ERK1/2 increased significantly when treated with palmitate for 12 h (Figure 4A) whereas the level of p-ERK1/2 decreased significantly and almost restored to the normal by pre-incubation with 2.5 μg/mL globular adiponectin. Taken together, these results suggested that adiponectin suppressed palmitate-induced apoptosis (Figure 2C, D) through reducing the activity of ERK1/2 signaling pathway. In order to further determine the role of the ERK1/2 in palmitate-induced H9c2 cells apoptosis, we used its inhibitor, 10 μM U0126 reference from [25]. We found that the level of p-ERK1/2 decreased significantly when treated with 10 μM U0126 in the presence of palmitate (Figure 4B) and the level of cleaved caspase-3 and cleaved PARP also decreased (Figures 4C, D). The results demonstrated that ERK1/2 signaling pathway was involved in palmitate-induced apoptosis in H9c2 cells and adiponectin partially inhibited palmitate-induced apoptosis through decreasing the level of phosphorylated ERK1/2.

### PI3K/Akt and ERK1/2 signaling pathway crosstalk plays a role in regulating adiponectin attenuated palmitate-induced apoptosis in H9c2 cells

**Figure 5:**
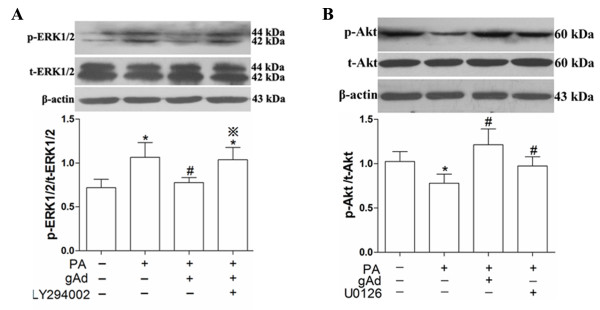
**Crosstalk between PI3K**/**Akt and ERK1**/**2 signaling pathway of palmitate**-**induced apoptosis in H9c2 cells.** Cells were pretreated with LY294002 (10 μM) or U0126 (10 μM) for 1 h, and then treated with 2.5 μg/mL globular adiponectin for 1 h before exposure to 150 μM palmitate for 12 h. Levels of p-ERK1/2 and t-ERK1/2 (**A**), p-Akt and t-Akt (**B**) were measured by immunoblot and quantitative analysis. These results were reported as mean ± S.E.M., * *P*< 0.05 versus control, # *P*< 0.05 verus PA and ※ *P*< 0.05 versus (PA + gAd).

Previous results indicated that 2.5 μg/mL globular adiponectin can attenuate palmitate-induced apoptosis in H9c2 cells through decreasing the level of p-ERK1/2, and simultaneously increasing the level of p-Akt. An interesting question was whether partial recovery of the activity of PI3K/Akt signaling pathway could lead to a decreased activity of ERK1/2 signaling pathway. Therefore, we further investigated the relationship between ERK1/2 and PI3K/Akt signaling pathway in adiponectin-mediated anti-apoptosis in H9c2 cells. Cells were exposed to 2.5 μg/mL globular adiponectin plus palmitate in the absence or the presence of PI3K inhibitor, 10 μM LY294002. Data showed that the level of p-ERK1/2 was increased dramatically (Figure 5A). Meanwhile, the level of p-Akt was also increased after cells were exposed to palmitate combined with ERK1/2 inhibitor 10 μM U0126 (Figure 5B). These results indicated that partial inhibition of PI3K/Akt signaling pathway resulted in activation of ERK1/2 signaling pathway, which attenuated effects of globular adiponectin on anti-apoptosis; and partial inhibition of ERK1/2 signaling pathway resulted in activation of PI3K/Akt signaling pathway and also attenuated palmitate-induced apoptosis in H9c2 cells.

## Discussion

Free fatty acids, such as saturated fatty acids are now recognized as significant contributors to lipotoxicity pathology including insulin resistance, type 2 diabetes, and cardiomyopathy [26]. Palmitate as a kind of saturated fatty acids can induce apoptosis in diverse cell types, such as cardiomyocytes [27]. Apoptosis or programmed cell death is basically cellular suicide which occurs after sufficient cellular damage. Caspase-3, a member of cysteine-aspartic proteases that play a central role in the execution of the apoptotic program, exists as an inactive 32-kDa proenzyme in normally. The cleavage within the 19-kDa fragment generates a p17-kDa subunit (termed p17) as an activity form. During apoptosis, caspase-3 cleaved the 116-kDa PARP protein to yield a 24-kDa DNA-binding fragment and an 89-kDa catalytic fragment [28]. In this study, our results showed that palmitate-induced apoptosis through increasing the activity of caspase-3 and PARP in H9c2 cells.

Adiponectin, an abundant circulating adipokine, is almost exclusively secreted from adipose tissue and exists in the range of 3–30 μg/mL in plasma. Although its physiological and pathological significance remains to be determined, numerous epidemiological studies have shown that the correlation between reduced adiponectin levels and increased morbidity/mortality of cardiovascular ischemic diseases and diabetes mellitus [29,30]. Conversely, a higher plasma adiponectin concentration is associated with a lower risk of ischemic heart disease [31]. Cardiomyocytes apoptosis is an important contributor to myocardial dysfunction and heart failure, so preventing cardiomyoytes apoptosis is an effective way to protect myocardial function. Recently some published *in vivo* studies demonstrated that adiponectin functioned as a cardioprotective molecule in myocardial ischemia-reperfusion injury [12,18,32]. The results of these research exhibited that exogenous adiponectin supplementation can significantly decrease myocardial apoptosis, infarct size and impaired cardiac function. More detailed *in vitro* studies regarding anti-apoptotic mechanisms of adiponectin have been performed in different cell types [33-35]. Although adiponectin also inhibited hypoxia/reoxygenation (H/R)-induced apoptosis through reducing cytochrome c release and decreasing the activity of caspase-3 in H9c2 cells [10], it is not clear that there is the effect of adiponectin on palmitate-induced apoptosis in H9c2 cells. In this study, our results showed that globular adiponectin inhibited palmitate-induced apoptosis in H9c2 cells through decreasing the activity of caspase-3 and PARP. Above data indicated that adiponectin might be a novel therapeutic molecule for anti-apoptosis in cardiomyopathy and myocardial damage.

Recently many studies showed that several signaling transduction pathways were shown to mediate both of pro- and anti-apoptosis effects in numerous tissues and cell types by adiponectin, such as (PI3K)/Akt signaling pathway, MAPK/ERK and AMPK.

Notably, PI3K/Akt signaling pathway has been shown to play a major role in the prevention of apoptosis [16], and acute activation of this signal pathway can promote both cardiomyocyte survival and function *in vitro* and *in vivo*[36]. Previous studies have shown that adiponectin can activate the Akt signaling pathway to promote pro-survival or anti-apoptosis in several cell types [35,37,38]. Here, our results showed that globular adiponectin can attenuate apoptosis induced by palmitate in H9c2 cells through decreasing the activity of caspase-3 and PARP. This effect was abolished by LY294002, a highly specific inhibitor of PI3K/Akt. This data suggested that activation of PI3K/Akt signaling pathway was necessary for adiponectin mediated inhibition of H9c2 cells apoptosis induced by palmitate.

ERK1/2/MAPK is a well-known taking part in a signal transduction cascade in response to extracellular stimuli, and plays an important role in cell proliferation, growth and cell death [17]. Research indicated that ERK1/2 signaling pathway would be activate by doxorubicin-induced apoptosis in H9c2 cells [39]. Adiponectin mediates activation of the ERK1/2 signaling pathway in several cell types [19,40]. Nevertheless, suppression of the activity of ERK1/2 signaling pathway by adiponectin has also been demonstrated [41]. Therefore, it seems that the effect of adiponectin on ERK1/2 signaling pathway is controversial. In this study, our results showed that the p-ERK1/2 was increased after palmitate-induced apoptosis in H9c2 cells, and globular adiponectin decreased the level of p-ERK1/2, and then inhibited palmitate-induced apoptosis in H9c2 cells through decreasing the activity of caspase-3 and PARP. In our results also showed that the level of p-ERK1/2 was increased after palmitate-induced apoptosis in H9c2 cells, and ERK1/2 inhibitor U0126 can decrease the level of p-ERK1/2, and then attenuate palmitate-induced apoptosis in H9c2 cells. These results suggest that activation of ERK1/2 signaling pathway may be one of the reasons for palmitate-induced apoptosis in H9c2 cells.

Our findings in this study showed that PI3K/Akt inhibitor LY294002, not only inhibited the activity of PI3K/Akt signaling pathway, blocked adiponectin’s inhibition of palmitate-induced apoptosis in H9c2 cells, but also increased the activity of ERK1/2 signaling pathway. Similarly, ERK1/2 inhibitor U0126 also reduced palmitate-induced apoptosis in H9c2 cells, increased the activity of PI3K/Akt signaling pathway and thus promoted cells survival. This crosstalk of ERK1/2 and PI3K/Akt were observed in other study [42]. These results suggested that ERK1/2 and PI3K/Akt signaling pathways maybe crosstalk regulates survival and apoptosis in H9c2 cells after treated with palmitate, but it regulates mechanism crosstalk in H9c2 cells require further investigation.

## Conclusions

Taken together, these results demonstrated that globular adiponectin can inhibit palmitate-induced apoptosis in H9c2 cells through inhibiting the activity of caspase-3, PARP and inhibiting the activation of the ERK1/2 signaling pathway while activating of the Akt signaling pathway. This study not only enriched mechanisms of adiponectin-mediated anti-apoptosis in cardiomyocytes, but also provided valuable evidence for identifying adioponectin as a candidate for the treatment of lipotoxicity cardiomyopathy.

## Competing interest

We declare that we have no competing interest.

## Authors' contributions

YL and CDW designed the research and wrote the manuscript, CDW and HYZ conducted the imaging experiment and measured cell viability or apoptosis. KSS, YQT, WD and WW contributed to the immunoblot. CDW and AYB analyzed data and performed statistical analysis. They have participated sufficiently in this work. All authors read and approved the final manuscript.
